# Glutathione S-transferase Mu 2 inhibits hepatic steatosis via ASK1 suppression

**DOI:** 10.1038/s42003-022-03251-w

**Published:** 2022-04-06

**Authors:** Yi Jin, Yanjie Tan, Pengxiang Zhao, Yu Guo, Shilin Chen, Jian Wu, Zhuqing Ren

**Affiliations:** 1 Key Laboratory of Agriculture Animal Genetics, Breeding and Reproduction of the Ministry of Education & Key Laboratory of Swine Genetics and Breeding of the Ministry of Agriculture, College of Animal Science, Huazhong Agricultural University, Wuhan, Hubei 430070 PR China; 2 Hubei Hongshan Laboratory, Wuhan, Hubei 430070 PR China

**Keywords:** Cell biology, Lipids, Metabolic disorders

## Abstract

Hepatic steatosis is the main characteristic of some liver metabolism diseases. However, unclear molecular mechanism of hepatic steatosis impedes the therapy of this hepatic steatosis. Glutathione-S-transferase mu 2 (*GSTM2*), as a member of phase II drug metabolizing enzymes (DMEs), regulates cellular antioxidant and detoxificant. *GSTM2* was highly up-regulated in hepatic steatosis tissues and high-fat diet (HFD) fed mice. Loss-of-function *GSTM2* mouse model demonstrated that *GSTM2* protected mice from excess fat accumulation. Mechanistically, *GSTM2* interacted with ASK1 and suppressed its phosphorylation and the activation of subsequent downstream p38-JNK signalling. Moreover, *GSTM2* overexpression in the liver effectively ameliorated hepatic lipid accumulation. Therefore, we identified *GSTM2* as an important negative regulator in progression of hepatic steatosis via both its detoxification/antioxidant and inhibition of ASK1-p38/JNK signalling. This study showed potential therapeutic function of the DME in progression of hepatic steatosis.

## Abstract (web summary)

Jin et al. investigate the underlying mechanisms of hepatic steatosis and show that Glutathione-S-transferase mu 2 (*GSTM2*), which is a drug metabolizing enzyme (DME), is upregulated in hepatic steatosis tissue from mice fed with a high fat diet. They show that *GSTM2* is a negative regulator of hepatic steatosis via both detoxification/antioxidant and inhibition of ASK1-p38/JNK signalling, which sheds light on its potential as a therapeutic target.

## Introduction

Hepatic steatosis, characterized by excessive accumulation of triglycerides (TGs) in hepatocytes, is considered the cause of non‐alcoholic fatty liver disease (NAFLD)^1–3^. Disordered lipid metabolism leads to the hepatic steatosis causing lots of excess fat accumulation in hepatocytes. High hepatic fat content is the important inducement of the impairment of redox imbalance and insulin resistance. However, the molecular mechanisms of hepatic steatosis occurrence and progression are poorly understood.

Drug-metabolising enzymes (DMEs) play an important role in scavenging the waste products of lipid metabolism and oxidative metabolism and maintaining homeostasis of liver^4,5^, consisted of groups of enzymes, such as glutathione S-transferases (GSTs). Several studies reported DMEs could respond to the hepatic steatosis by protecting hepatocytes from free radicals^6,7^, additionally, GSTs are always considered as the scavenger of reactive oxygen species. Among other GSTs, glutathione *S*-transferase M2 (*GSTM2*) seems to be different. Previous researches from Huenchuguala et al. reported that they found cells with *GSTM2* knockdown accumulated more lipid droplets^8–11^. Moreover, *GSTM2* was highly up-regulated in high-fat diet mouse liver^6,7^. These studies suggested *GSTM2* regulated lipid metabolism specially beyond detoxification/antioxidant, the basic functions of GSTs.

In the present study, the close association between *GSTM2* knockout and hepatic steatosis was examined by using *GSTM2*-null mice. We demonstrated that *GSTM2* protects against hepatic steatosis by inhibiting excess fat accumulation by interacting with and suppressing activation of apoptosis signal-regulating kinase1 (*ASK1*) and subsequent p38-JNK signalling, besides its antioxidant capacity. Moreover, *GSTM2* overexpression reversed methionine choline-deficient diet (MCDD)-induced steatosis. Thus, we identified *GSTM2* as an important negative regulator in hepatic steatosis progress.

## Results

### GSTM2 was upregulated in hepatic tissues of mice fed HFD and MCDD

**Fig. 1:**
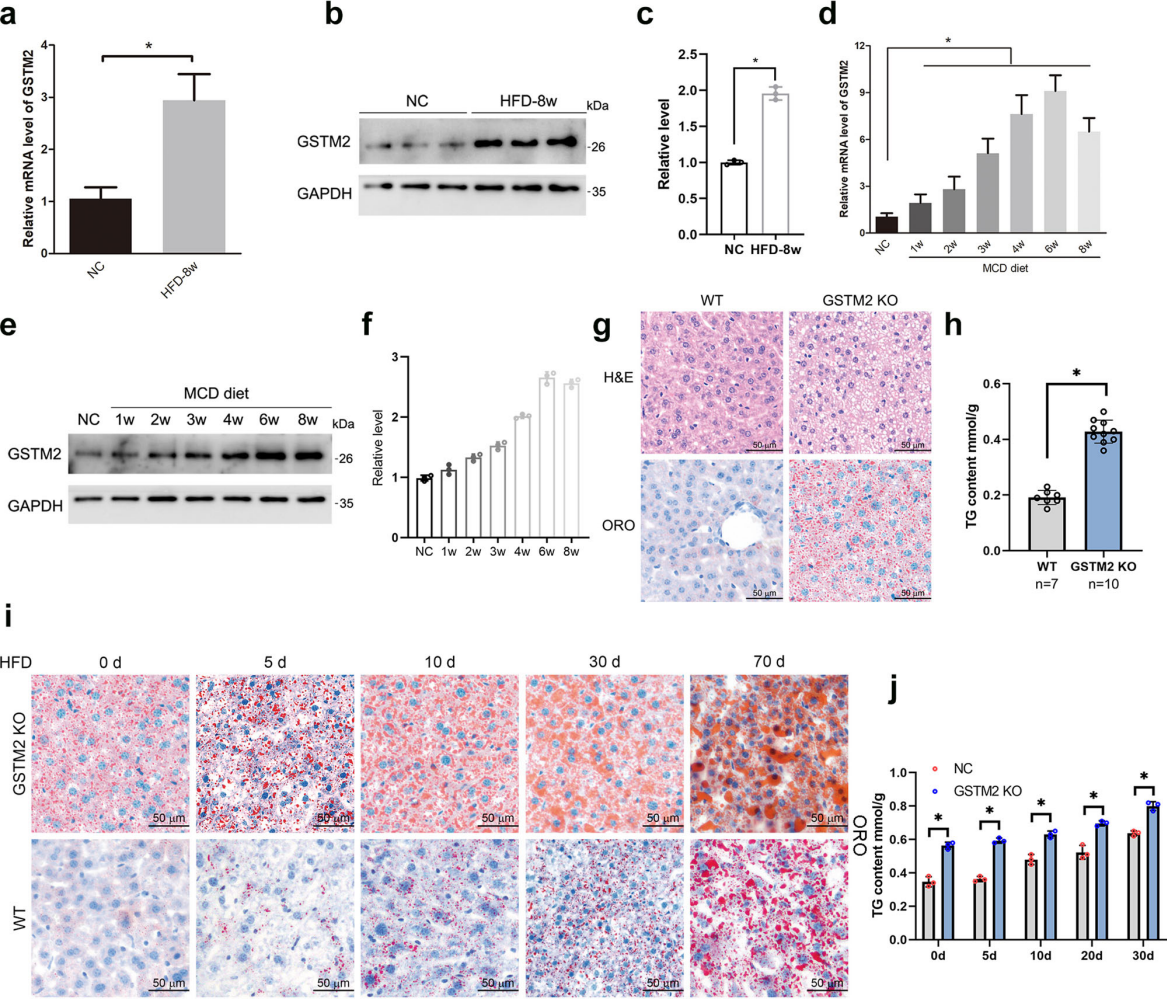
Loss-of-function of GSTM2 promoted hepatic fat storage. **a**–**c** GSTM2 mRNA and protein expression in liver samples of 8-week HFD fed mice (*n* = 3) and 8-week chow diet fed mice (*n* = 3); *p < 0.05. **d**–**f** GSTM2 mRNA and protein expression in liver samples of MCDD fed mice and corresponding control mice; *p < 0.05. GAPDH served as the reference gene. **g** Haematoxylin and eosin (HE) and Oil red O staining analysis of liver samples of GSTM2 knockout (KO) and control mice; bar = 20 μm. **h** Hepatic TG concentrations of WT (*n* = 7) and GSTM2 KO (*n* = 10) mice were detected; ***p < 0.001. **i** Oil red O analysis of liver samples of GSTM2 KO and control mice challenged with HFD at 0 d, 5 d, 10 d, 20 d, and 30 d. **j** Hepatic TG concentrations of WT and GSTM2 KO mice with high-fat diet treatment at 0 d, 5 d, 10 d, 20 d, and 30 d; *p < 0.05. bar, 50 μm.

The high-fat diet and methionine choline deficient diet were used to make hepatic steatosis. We validated the expression pattern of *GSTM2* in the HFD and MCDD mouse models. The level of *GSTM2* mRNA and protein were detected. *GSTM2* was significantly up-regulated in mice fed HFD (Fig. 1a–c) and MCDD (Fig. 1d–f).

### GSTM2 deletion strongly promotes hepatic fat accumulation

To investigate the function of *GSTM2* in the progression of hepatic steatosis, we generated a *GSTM2* knockout (*GSTM2* KO) mouse (Supplementary Fig. 1a, b). Hepatic histomorphology and TG content detection showed that *GSTM2* KO mice had higher hepatic fat content (Fig. 1g, h). Moreover, there was no significant difference in the ratio of liver weight to body weight between *GSTM2* KO and WT mice (Supplementary Fig. 1c, d). We also investigated the fat content in white adipose and skeletal muscle tissues via Oil Red O staining. More intramuscular fat was observed in *GSTM2* KO mice, whereas no significant difference was found in white adipose tissue between KO and WT mice (Supplementary Fig. 1e, f). To further investigate the function of *GSTM2* in hepatic lipid metabolism, we challenged the mice with HFD. KO mice accumulated more fat at a faster rate than WT mice (Fig. 1i, j, Supplementary Fig. 1g). Moreover, the hepatic alanine aminotransferase (ALT) level was higher in KO mice, while the γ-glutamyltranspetidase (GGT) level was not changed (Supplementary Fig. 1h, i). We next challenged the mice with 0.2 mM oleic acid medium (dissolved by BSA in 0.9% NaCl solution) to investigate the effect of *GSTM2* on the rapid formation of LDs. Hepatic morphology analysis showed that *GSTM2*-KO-livers formed more LDs than WT livers (Supplementary Fig. 1j, k). These results suggest that *GSTM2* plays an important role in excess hepatic fat accumulation.

### GSTM2 overexpression suppresses hepatic steatosis

**Fig. 2:**
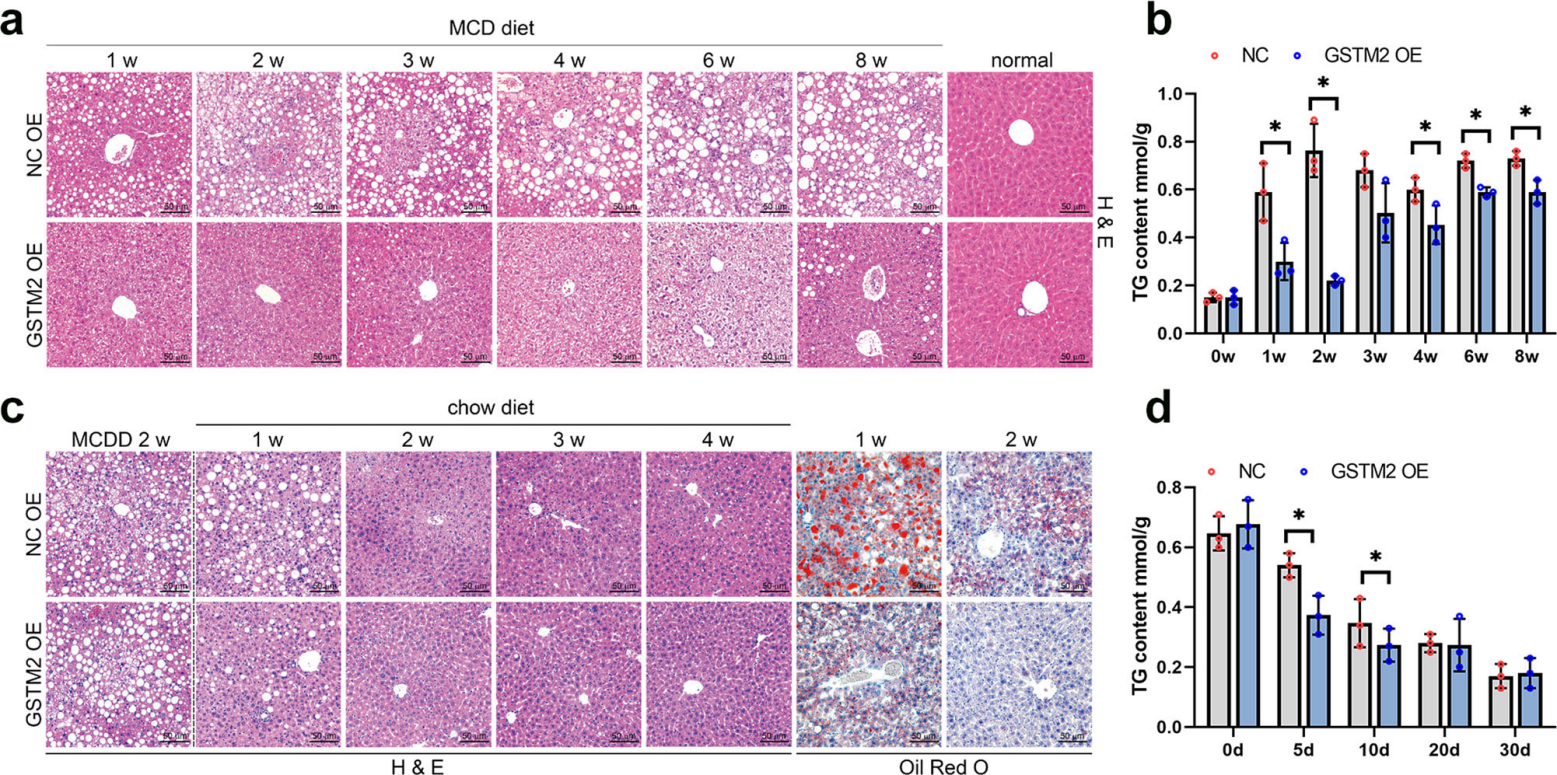
GSTM2 overexpression protected against hepatic fat storage. **a** HE staining analysis of liver samples of GSTM2 overexpression (OE) and control mice challenged with MCDD at 1 w, 2 w, 3 w, 4 w, 6 w, and 8 w. **b** Hepatic TG concentrations of GSTM2 OE and control mice with MCD diet treatment at 0 w, 1 w, 2 w, 3 w, 4 w, 6 w, and 8 w; *p < 0.05. **c** Oil red O staining analysis of liver samples of GSTM2 OE and control mice fed MCDD for 2 w and then chow diet. **d** Hepatic TG concentrations of corresponding mice in (**c**); *p < 0.05. bar, 50 μm.

Although *GSTM2* was up-regulated in mice with hepatic steatosis, we also overexpressed this protein in mouse model fed with MCDD (Supplementary Fig. 2a). Western blot analysis showed that *GSTM2* was highly expressed in liver tissues (Supplementary Fig. 2b). As the feeding time increased, control mice accumulated much more hepatic fat, while GSTM2 overexpression suppressed hepatic fat accumulation (Fig. 2a). Control mice showed significant hepatic steatosis after 1 week of MCDD feeding, whereas mice with *GSTM2* overexpression showed this effect at 4 weeks. Additionally, *GSTM2*-overexpressing mice showed mild LD accumulation in the first 3 weeks (Fig. 2b). Because fibrosis is the key signal of steatosis aggravation, we further detected the fibrosis level in liver tissues. Masson staining analysis indicated that control mice had significant hepatic fibrosis compared to mice with *GSTM2* overexpression (Supplementary Fig. 2c, d). A significant degree of fibrosis was observed in liver tissues of control mice at 3 weeks, whereas *GSTM2*-overexpressing mice showed mild fibrosis at 8 weeks (Supplementary Fig. 2c, d). Moreover, we detected the marker genes of fibrosis *α-SMA* and *CoL1A1*. The results showed the expression of *α-SMA* and *CoL1A1* was up-regulated at 8 weeks significantly (Supplementary Fig. 2e). These results suggest that *GSTM2* overexpression inhibits hepatic steatosis development. We subsequently performed a rescue test. A GSTM2 overexpression vector was transfected into mice fed MCDD for 2 weeks, which was then changed to a chow diet (Supplementary Fig. 2f). Overexpression efficiency was detected by Western blotting (Supplementary Fig. 2g). As expected, *GSTM2* overexpression promoted the recovery of hepatic steatosis compared to control (Fig. 2c). Although both *GSTM2*-overexpressing mice and control mice showed good recovery of hepatic steatosis at 2 weeks, *GSTM2*-overexpressing mice showed lower hepatic fat content at 1 week (Fig. 2d). The rescue experiment suggests that *GSTM2* may be a useful therapeutic target for hepatic steatosis.

### GSTM2 modulates size and number of LDs in HepG2 cells

**Fig. 3:**
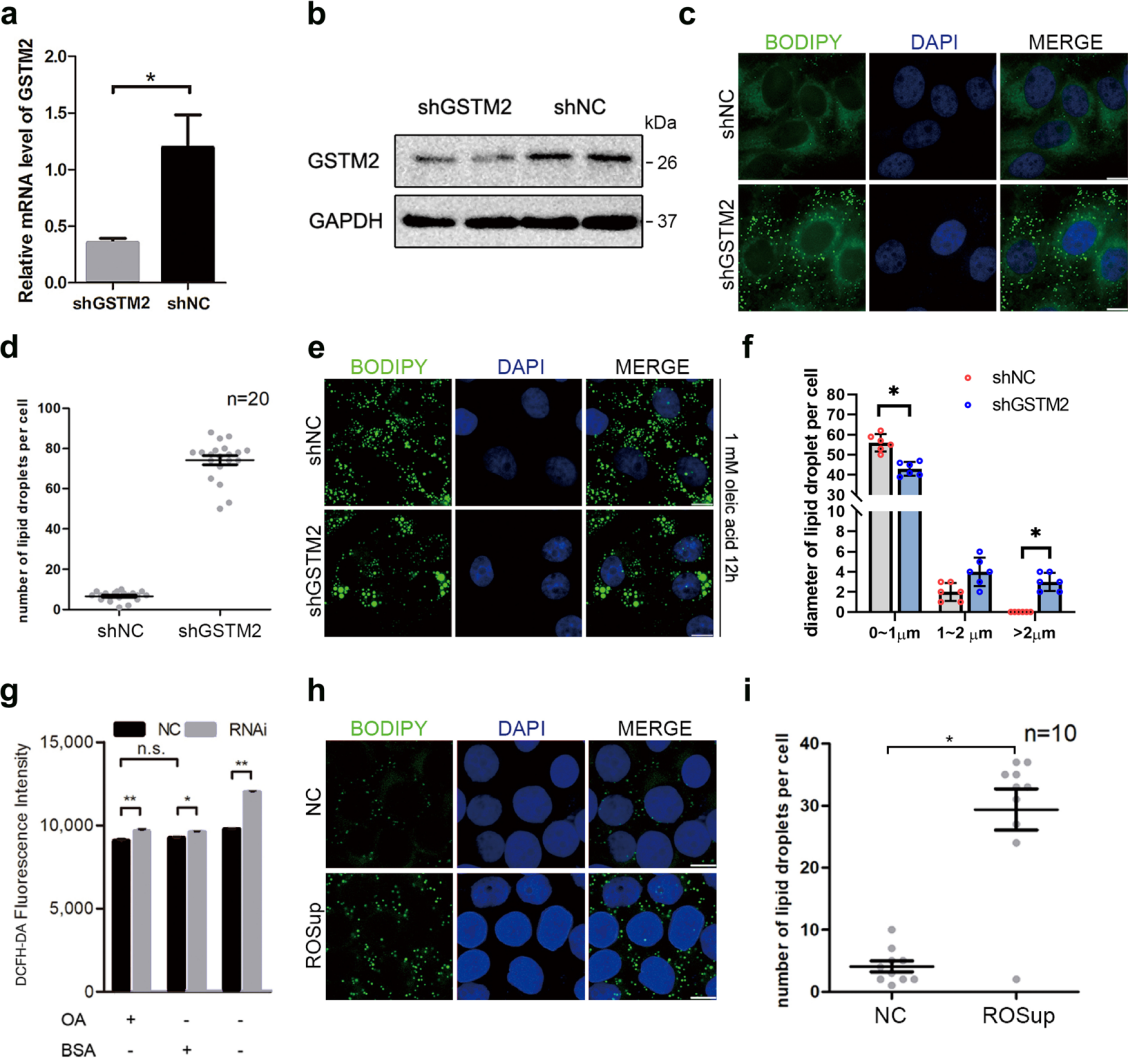
GSTM2 modulated cellular lipid droplets accumulation via regulating cellular ROS level. **a**, **b** Expression level of GSTM2 was detected in shGSTM2-HepG2 cells and corresponding control cells. **c** More LDs were observed in shGSTM2-HepG2 cells. **d** Statistics of LD number in shGSTM2-HepG2 cells and shNC-HepG2 cells. **e** More larger LDs were found in shGSTM2-HepG2 cells treated with 1 mM oleic acid medium for 12 h compared to control cells. **f** Statistics of LD diameter in shGSTM2-HepG2 cells and shNC-HepG2 cells treated with 1 mM oleic acid medium for 12 h compared to control cells. **g** Cellular ROS level was detected in shGSTM2-HepG2 and shNC-HepG2 cells in the presence or absence of oleic acid medium; *p < 0.05, **p < 0.01. **h** More LDs were observed in cells treated with ROSup compared to control cells. **i** Statistics of LD number in cells treated with ROSup and control cells. Bar, 10 μm.

To further investigate the potential regulatory mechanism, we generated a *GSTM2*-knockdown stable cell line (shGSTM2-HepG2) that expressed less *GSTM2* mRNA and protein than control cells (shNC-HepG2) (Fig. 3a, b). Interestingly, shGSTM2-HepG2 cells contained more LDs (~5 to 6 fold) in normal culture than shNC-HepG2 cells (Fig. 3c, d) and formed more and larger LDs (>2 μm) under fatty acid (FA)-rich (200 μM oleic acid medium) culture conditions (Fig. 3e, f).

### Effects of GSTM2 on LD content do not absolutely depend on regulating ROS level

Because *GSTM2* has antioxidant activity (as described in the Introduction), the potential effect of ROS on cellular lipid formation should be examined. We determined ROS levels in shGSTM2-HepG2 and shNC-HepG2 cells in normal and FA-rich culture medium, respectively. As expected, the ROS level was significantly increased (~15–20%) by *GSTM2* knockdown (*p* < 0.01) (Fig. 3g). We further investigated whether the increased cellular ROS levels affected the LD content by treating cells with an ROS activator (ROSup). As predicted, more cellular LDs (~2 to 3 fold compared to control) were observed in ROSup-treated cells (Fig. 3h, i). Indeed, the higher ROS level increased the LD number, but only approximately 30 LDs were observed in ROSup-treated cells (Fig. 3i), which was much lower than the number detected in shGSTM2-HepG2 cells. Therefore, the increase in ROS did not greatly alter the cellular LD content.

We next investigated the changes in LD formation and expression of breakdown-related genes at different ROS levels. The expression of *PLIN2, PLIN5, and ATGL* was significantly increased by ROSup treatment (*p* < 0.05) (Supplementary Fig. 3a). *PLIN2, PLIN3*, and *SREBF1* increased and *PCYT1A* decreased significantly following *GSTM2* knockdown (*p* < 0.05) (Supplementary Fig. 3b). As expected, *GSTM2* overexpression significantly reduced the cellular ROS level (Supplementary Fig. 3c); therefore, we examined gene expression changes after *GSTM2* overexpression. In contrast to the results of *GSTM2* knockdown, *PLIN2* and *SREBF1* were down-regulated and *PCYT1A* was up-regulated, showing significant differences (*p* < 0.05) (Supplementary Fig. 3d). Because *N*-acetylcysteine (NAC) is an effective ROS scavenger (Supplementary Fig. 3e), we investigated whether a reduced ROS level would alter the LD content by treating cells with 1 mM NAC. Interestingly, NAC did not significantly change the number of LDs in either normal or FA-rich cultural medium (Supplementary Fig. 3f, g). To evaluate the contribution of the *GSTM2* oxidative ability in promoting LD formation, we added ROS scavenger (NAC) in GSTM2 knockdown cells and detected the LD number. The result showed that NAC did suppress the LD number induced by *GSTM2* knockdown (Supplementary Fig. 3h). However, the LD number in NAC treatment group was still more than NC group (Supplementary Fig. 3h). These results suggest that modulating ROS levels by *GSTM2* is not the only approach for regulating the cellular lipid content.

### GSTM2 modulates p38-JNK signalling by inhibiting ASK1 activity

**Fig. 4:**
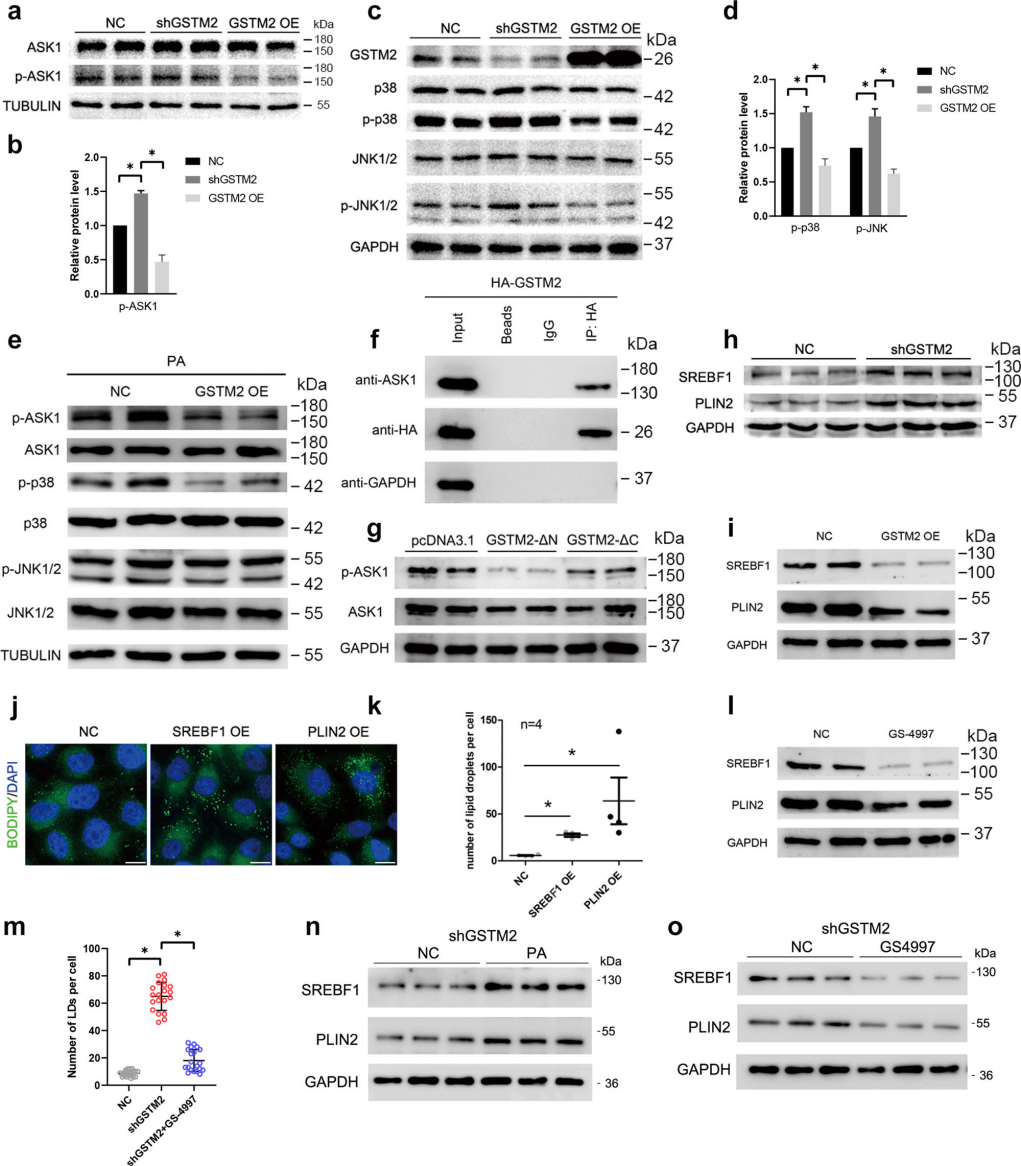
GSTM2 regulated lipid droplet accumulation via ASK1-p38/JNK signalling. **a**, **b** Phosphorylation of ASK1 was up-regulated by GSTM2 KD and down-regulated by GSTM2 OE. **c**, **d** p-p38 and p-JNK levels were up-regulated by GSTM2 knockdown (KD) and down-regulated by GSTM2 OE. **e** GSTM2 OE down-regulated ASK1-p38/JNK signalling in the presence of palmitic acid treatment. **f** GSTM2 interacted with ASK1 by co-IP detection. **g** GSTM2 C-terminal, not N-terminal, down-regulated ASK1 activity. **h** SREBF1 and PLIN2 expression level was up-regulated by GSTM2 KD. **i** SREBF1 and PLIN2 expression level was down-regulated by GSTM2 OE. **j** More LDs were observed in cells with SREBF1 or PLIN2 overexpression. **k** Statistical analysis of number of LDs in cells with SREBF1 or PLIN2 overexpression. **l** Expression levels of SREBF1 and PLIN2 were down-regulated by GS-4997 treatment (ASK1 activity inhibitor). **m** Statistical analysis of number of LDs in shGSTM2 cells and shGSTM2 cells treated with GS-4997. **n** WB detection of SREBF1 and PLIN2 in shGSTM2 cells treated with PA. **o** WB detection of SREBF1 and PLIN2 in shGSTM2 cells treated with GS-4997. Bar, 10 μm.

ASK1-p38/JNK signalling plays an important role in NAFLD development, thus we investigated whether this signalling was affected by *GSTM2* expression. The phosphorylation level of ASK1 was regulated by GSTM2. The p-ASK1 level was increased by GSTM2 knockdown and decreased by GSTM2 overexpression (Fig. 4a, b). Moreover, *GSTM2* knockdown and overexpression had different effects on p38-JNK signalling. The phosphorylation levels of p38 and JNK were increased by *GSTM2* knockdown, but decreased by GSTM2 overexpression (Fig. 4d). We further examined the effect of *GSTM2* on ASK1-p38/JNK signalling in the presence of palmitic acid (PA), an ASK1 activator. As expected, *GSTM2* overexpression reduced ASK1 signalling activation (Fig. 4e). AKS1 signalling was activated and enhanced in liver tissues with the development of NAFLD^12^, which leads to severe inflammation and fibrosis. This result suggests that *GSTM2* has a potential therapeutic function in NAFLD.

### C-terminus of GSTM2 inhibits ASK1 phosphorylation by binding to ASK1

We then investigated the mechanism of GSTM2 regulation of ASK1 activity. Previous studies reported that the C-terminus of GSTM1 binds to ASK1 and suppresses the phosphorylation of ASK1^13,14^. Thus, we compared the amino acid sequences and 3D structures of these two proteins. GSTM1 and GSTM2 shared highly similar sequences and structures (Supplementary Fig. 4a, b), with >90% similarity. Moreover, another study reported that GSTM2 binds to ASK1^15^. We next examined whether GSTM2 interacts with ASK1 by conducting a co-immunoprecipitation assay. The result indicated that GSTM2 binds to ASK1 (Fig. 4f). We then investigated the structural details of this binding interaction. GSTM2 contains two main functional domains, GST-binding (N-terminal) and C-terminal domains (Supplementary Fig. 4c). Overexpression of the C-terminus of GSTM2 decreased the p-ASK1 level, whereas N-terminal overexpression did not (Fig. 4g).

### GSTM2 regulates expression levels of SREBF1 and PLIN2

SREBF1 and PLIN2 play important roles in cellular lipid metabolism. A previous study reported that SREBF1 is regulated by p38-JNK signalling^16,17^ and PLIN2 expression is regulated by SREBF1. We detected the effects of *GSTM2* on the expression of these two genes. As expected, the expression levels of SREBF1 and PLIN2 were increased by *GSTM2* knockdown and decreased by *GSTM2* overexpression (Fig. 4h, i). We confirmed the effects of these two genes on cellular LD content by overexpressing *SREBF1* and *PLIN2*. More LDs were observed in cells with both *SREBF1* and *PLIN2* overexpression (Fig. 4j, k). Next, GS-4997, a specific ASK1 inhibitor, was used to confirm that the expression of SREBF1 and PLIN2 is regulated by ASK1-p38/JNK signalling. SREBF1 and PLIN2 expression was decreased in cells treated with GS-4997 (Fig. 4l).

To further validate the role of ASK1 in *GSTM2* knockdown cells, we detected LDs number in *GSTM2* knockdown cells treated with GS-4997. The result showed that GS-4997 greatly decreased LDs number compared to *GSTM2* knockdown cells (Fig. 4m). We further detected the protein level of SREBF1 and PLIN2 in *GSTM2* knockdown cells treated with PA or GS-4997. The results indicated that PA increased the expression level of SREBF1 and PLIN2 (Fig. 4n), whereas GS-4997 suppressed the expression level of SREBF1 and PLIN2 (Fig. 4o). The results indicated that *GSTM2* regulated SREBF1 and PLIN2 expression by affecting ASK1 activity.

### GSTM2 modulates growth of LDs via ASK1-p38/JNK signalling

**Fig. 5:**
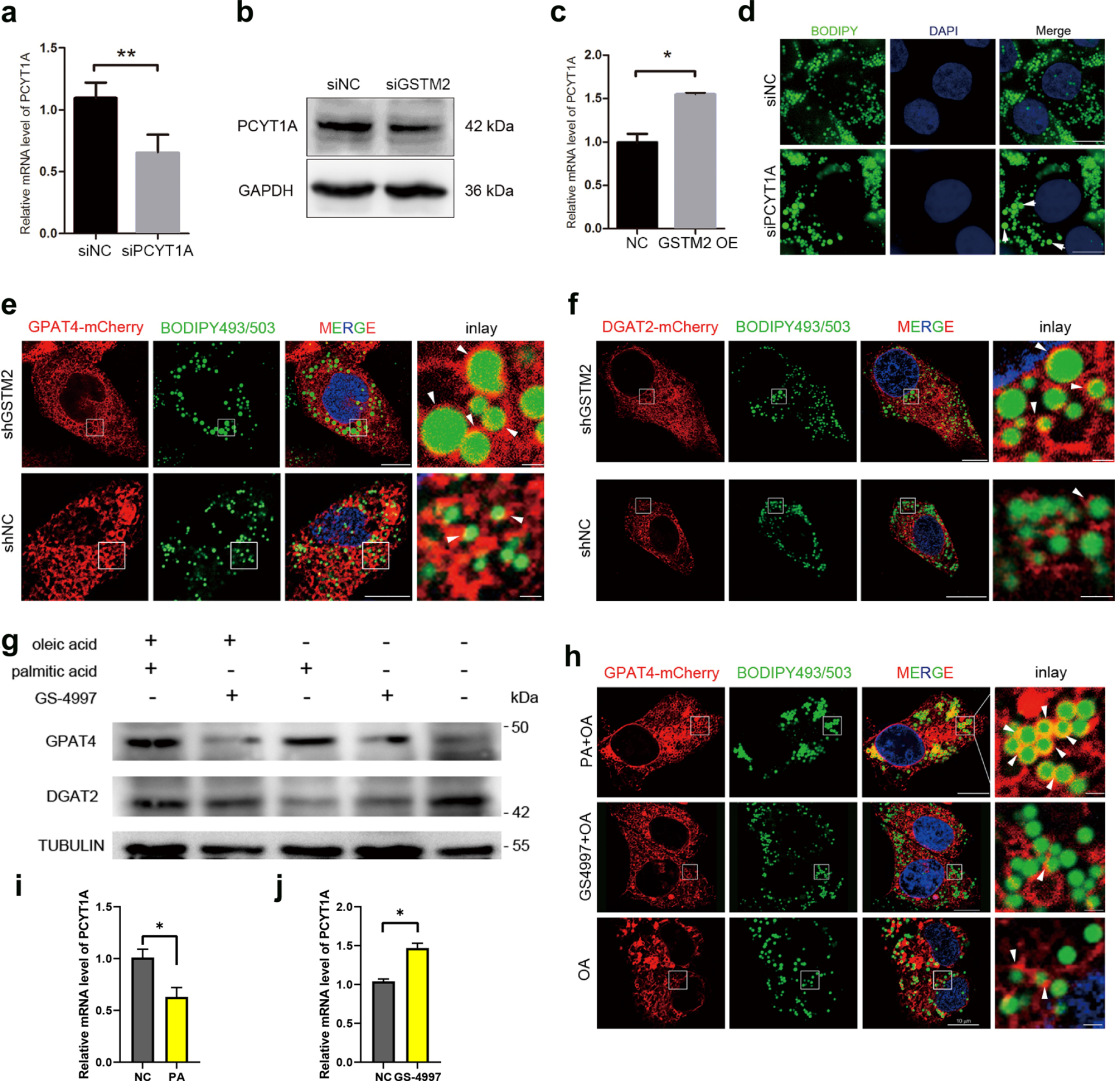
GSTM2 modulated lipid droplet growth via ASK1 signalling. **a**–**c** PCYT1A expression level was down-regulated by GSTM2 KD and up-regulated by GSTM2 OE; *p < 0.05, **p < 0.01. **d** PCYT1A KD induced LD expansion. **e**, **f** GSTM2 affected recruitment of GPAT4 and DGAT2 on LD surface (in the presence of oleic acid medium), bar, 10 μm, 1 μm (inlay). **g** Expression level of GPAT4 and DGAT2 was regulated by treatment of palmitic acid (ASK1 activator) and GS-4997 (ASK1 activity inhibitor). **h** Recruitment of GPAT4 on LD surface was regulated by palmitic acid or GS-4997 treatment, bar, 10 μm, 1 μm (inlay). **i**, **j** qPCR detection of PCYTA1 expression with PA or GS-4997 treatment respectively.

The growth of LDs is important in increasing the cellular capacity for lipid storage. Previous studies demonstrated that *PCYT1A*, *GPAT4* and *DGAT2* are responsible for increasing the size of LDs^18–20^. The expression level of PCYT1A was positively correlated with *GSTM2* expression, which was confirmed by *GSTM2* knockdown and overexpression assays (Fig. 5a–c). Furthermore, *PCYT1A* knockdown induced the formation of larger LDs in FA-rich cultural medium (Fig. 5d). In addition to regulating LD growth, *PCYT1A* is related to LD fusion or coalescence. We examined the events involved in LD contact using a living cell station. The supplementary movie shows that *GSTM2* knockdown induced more LD–LD contact events (Supplementary movie S1) than in the control (Supplementary movie S2).

DGAT2 and GPAT4 targeting the surface of LDs is important for LD expansion. As described above, *GSTM2* modulated the size of LDs. We investigated whether this regulation was dependent on modulation of DGAT2 and GPAT4 targeting the LD surface. We found that more DGAT2 and GPAT4 were recruited to the LD surface by *GSTM2* knockdown (Fig. 5e, f). Next, we investigated the effect of ASK1-p38/JNK signalling on this progression. The expression level of DGAT2 and GPAT4 was detected in the presence of PA (ASK1 activator) or GS-4997 (ASK1 inhibitor) in normal or FA-rich cultural medium. The expression level of these protein was up-regulated by ASK1 signalling activation and down-regulated by ASK1 signalling suppression (Fig. 5g). Furthermore, recruitment of GPAT4 to the LD surface was enhanced by PA treatment and reduced by GS-4997 treatment (Fig. 5h). We further detected the effect of ASK1 activity on PCYT1A expression. The expression of PCYT1A was decreased by PA treatment, whereas was up-regulated by GS-4997 treatment (Fig. 5i, j, *p* < 0.05).

### GSTM2 KO induced hepatic fat accumulation was suppressed by GS-4997 in vivo

**Fig. 6:**
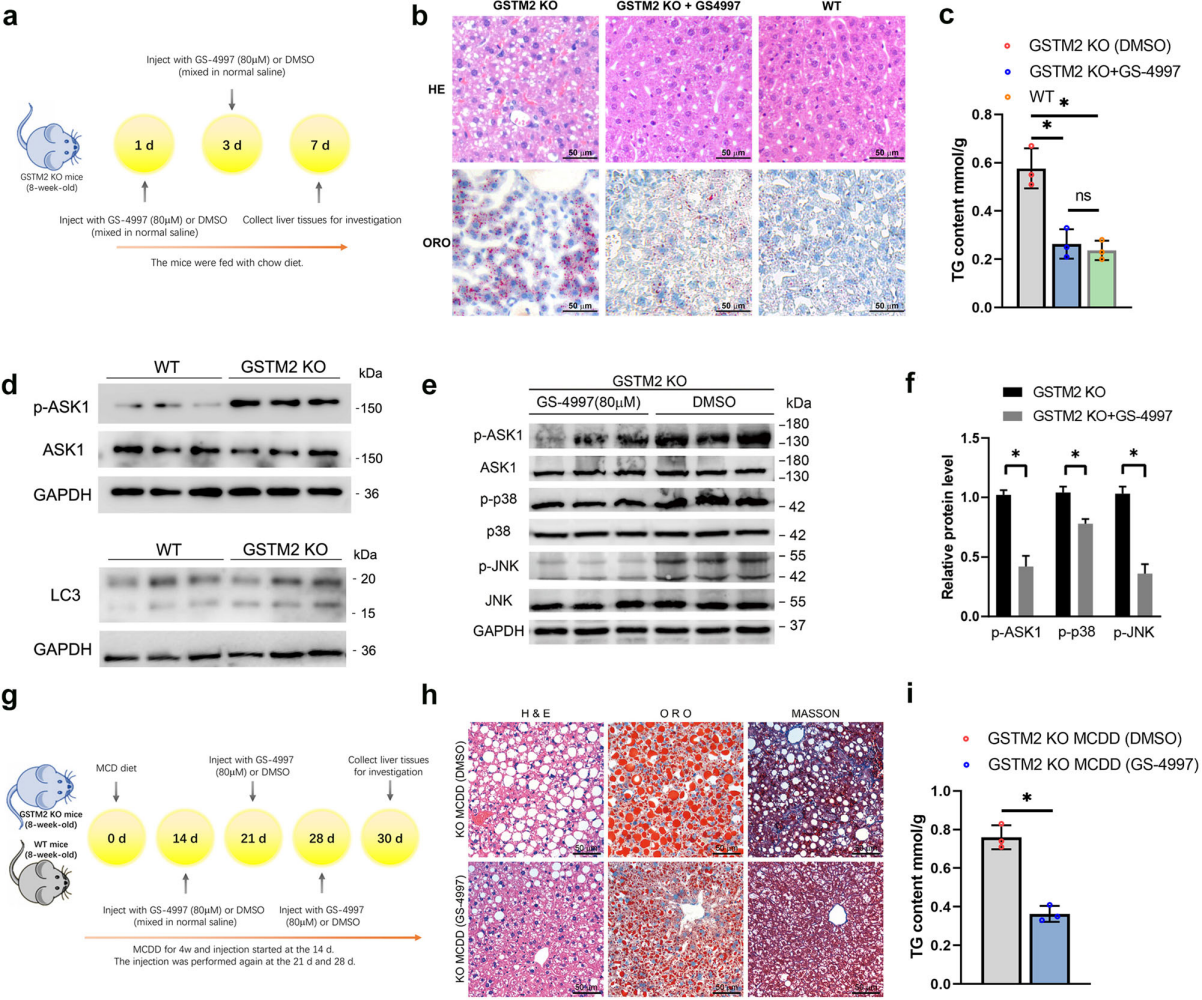
GSTM2 KO induced hepatic fat accumulation was suppressed by GS-4997 in vivo. **a** The GSTM2 KO mice were fed with chow diet and treated with GS-4997 or DMSO at day 1 and 3, then the liver tissues were collected for further analysis. **b** GSTM2 KO mice were treated with GS-4997 (ASK-1 inhibitor) by intraperitoneal injection. Hepatic fat was detected by HE and ORO staining. **c** TG content examination of WT mice fed MCDD with GS-4997 or DMSO treatment. **d** WB detection of expression level of p-ASK1 and LC3 in GSTM2 KO mice liver. **e** Investigation of protein levels of ASK1 signalling pathway in GSTM2 KO mice with GS-4997 or control, DMSO treatment by Western blot. **f** Fray value analysis of (**e**) by ImageJ software. **g** The WT and GSTM2 KO mice were fed with MCD diet for one month. The mice were treated with GS-4997 or DMSO at day 14, 21 and 28, and then the liver tissues were collected for further Analysis. **h** GSTM2 KO mice were fed MCDD and treated with GS-4997 or DMSO by intraperitoneal injection. Then hepatic fat and fibrosis level were detected by HE, ORO and Masson staining. **i** TG content examination of GSTM2 KO mice fed MCDD with GS-4997 or DMSO treatment. bar, 50 μm.

We then investigated whether GS-4997 treatment could rescue the hepatic steatosis induced by *GSTM2* knockout. GSTM2 KO mice were treated with 80 μM GS-4997 by intraperitoneal injection, and control mice were treated with an equal amount of DMSO solution (Fig. 6a). Then live tissues were collected and examined. We found that the GS-4997 treatment group showed less hepatic fat accumulation compared to the control group (Fig. 6b), and the TG content detection also supported this result (Fig. 6c). The results indicated that GS-4997 greatly suppressed the increase of TG accumulation induced by *GSTM2* KO. Moreover, we detected the p-ASK1 level in *GSTM2* KO mice, which showed the ASK1 was activated by *GSTM2* deletion (Fig. 6d). Previous study suggested that autophagy affected the TG utilization in liver^21,22^. We detected the expression level of LC3 in liver of *GSTM2* KO mice. The result showed the expression level of LC3-I and LC3-II was not changed in *GSTM2* KO mice (Fig. 6d). We then investigated the protein level of ASK1-p38/JNK signalling pathway. As expected, the levels of p-ASK1, p-p38 and p-JNK in the GS-4997 treatment group were decreased significantly (*p* < 0.05) (Fig. 6e, f).

Subsequently, we detected whether GS-4997 treatment could block the progress of hepatic steatosis in KO and WT mice with MCD diet. The mice were fed with MCD for one month. During that time, GS-4997 was injected at 14 d, 21 d and 28d (Fig. 6g). Then live tissues were collected for further investigation. Histological examination showed that GS-4997 treatment decreased lipid accumulation in WT mice with MCDD (Supplementary Fig. 5a); additionally, the TG content in live tissues was also decreased compared to the DMSO treatment group (Supplementary Fig. 5b). The results of Western blot showed that p-ASK1, p-p38 and p-JNK protein levels were decreased significantly by GS-4997 injection in WT mice with MCDD (*p* < 0.05) (Supplementary Fig. 5c). Furthermore, GS-4997 also inhibited fat accumulation in liver tissues of KO mice with MCDD (Fig. 6h). The TG content of the GS-4997 treatment group was decreased (*p* < 0.05) (Fig. 6i). Moreover, the protein level of p-ASK1, p-p38 and p-JNK was decreased in KO mice with MCDD by GS-4997 treatment (*p* < 0.05) (Supplementary Fig. 5d). Besides detecting the effect of GS-4997 treatment on hepatic fat accumulation, we also investigated the degree of hepatic fibrosis of the samples. As expected, GS-4997 suppressed hepatic fibrosis in both WT and KO mice with MCDD (Fig. 6g, Supplementary Fig. 5a), as detected by Masson staining. These results support that *GSTM2* knockout induced hepatic steatosis resulting from ASK1 signalling activation and can be rescued by treatment with GS-4997, an ASK1 inhibitor, in vivo.

## Discussion

The risk of hepatic steatosis is greatly increased by an unhealthy lifestyle and excess energy intake. The regulatory mechanism of steatosis is unclear. Here, we showed that *GSTM2* negatively regulated hepatic steatosis. *GSTM2* inhibited p38 and JNK signalling by suppressing activation of the upstream kinase ASK1. More importantly, we showed that overexpression *GSTM2* had a therapeutic effect on hepatic steatosis. Mechanistically, *GSTM2* modulated the formation of hepatic fat mainly by regulating the number and size of cellular LDs.

Few studies have evaluated the relationship of DME and hepatic steatosis. Our study demonstrated that the hepatic phase II DME, *GSTM2*, was involved in the development of hepatic steatosis. A previous study showed that hepatic steatosis increased the toxicity of hepatic drugs by down-regulating the expression of some DMEs^23^. Moreover, many studies showed that hepatic steatosis increased the risk of drug-induced liver disease^24–27^. hepatic steatosis may impair hepatic self-protection. Previous studies reported that *GSTM2* is highly up-regulated in NAFLD or in an HFD-induced fatty liver mice model (by more than 4-fold)^6,7^, but the mechanism was not evaluated. As a hepatic functional gene, up-regulation of GSTM2 may function in hepatic self-protection. Knockout and overexpression of *GSTM2* in a mouse model revealed its regulatory function in the development of hepatic steatosis. *GSTM2* KO enhanced the development of hepatic steatosis. Although *GSTM2* overexpression did not block the development of hepatic steatosis, it delayed the progression and reduced lipid accumulation and fibrosis progression. These results suggest that a liver with steatosis protects itself against disease injury by up-regulating *GSTM2*.

In addition to drug-induced toxicity, ROS is another metabolic by-product that can induce potential cytotoxicity. Many studies have demonstrated a relationship between ROS and lipid accumulation, particularly in fatty liver disease and hepatoma carcinoma^28–30^. Superoxide dismutase 1 (SOD1)-knockout mice showed higher hepatic fat storage, and more LDs were observed in SOD1 KO hepatocytes^17,31^. We also found that ROSup treatment induced greater LD formation in HepG2 cells in this study. As an important cellular antioxidant, glutathione is responsible for eliminating ROS. Therefore, GSTM2 can promote ROS degradation by enhancing the activity of glutathione, as demonstrated by our results. We predicted that *GSTM2* could alleviate ROS-induced neutral lipid accumulation and increase the fat content in NAFLD. Interestingly, in a previous study glomerulonephritis was treated by injecting GSTM2-transduced mesenchymal stem cells. The researchers found that GSTM2 expression significantly reduced oxidation and inflammation in the disease tissues and greatly ameliorated glomerulonephritis^32^. In our study, we observed a lower occurrence of fibrosis in GSTM2-overexpressing mice than in control mice. This phenotype is attributed to the function of *GSTM2* which ameliorates cellular stress and inflammation. We detected the antioxidant expression level in the *GSTM2* KO mice model and found that most antioxidants including *SOD1, CAT*, and *GPX1* were down-regulated. The higher level of ROS and inflammation may impair the function and expression of these antioxidants. Notably, GSTM1 shows high similarity in sequence and structure (>90%) to *GSTM2*, and Bhattacharjee et al. reported that GSTM2 can play a compensatory role in the absence of GSTM1^33^. Indeed, GSTM1 has a similar function as *GSTM2*, but several single-nucleotide polymorphisms may lead to gene inactivation. Moreover, no inactivating mutation of *GSTM2* has been found in human or house mouse. We considered that GSTM2 was likely be more stable than *GSTM1* from a genetic perspective, and therefore, up-regulating *GSTM2* may be a better choice for inducing protective effects.

We demonstrated that *GSTM2* suppressed the progression of hepatic steatosis by inhibiting ASK1-p38/JNK signalling. This signalling has been reported to play an important role in the development of NAFLD and NASH. ASK1, also known as MAP3K5, activates the downstream p38-JNK1/2 signalling pathway, thereby promoting inhibition of lipid and glucose metabolism^34–36^ and driving a strong inflammatory response^37^. ASK1 is currently considered as a target site for NASH therapy^38,39^, and ASK1 signalling is abnormally active in the liver of NAFLD and obese individuals. Inhibition of ASK1 activity significantly inhibits the development of NASH. For example, selonsertib (GS-4997) is a highly selective and potent ASK1 inhibitor with potential anti-inflammatory, anti-tumour, and anti-fibrotic activities^40^. Clinical trial results of the patient showed that GS-4997 has a good therapeutic effect on NASH. However, GS-4997 completely inhibits the activity and normal function of ASK1 with potential side effects^41,42^. In our study, hepatic *GSTM2* was found to suppress steatosis by inhibiting ASK1-p38/JNK signalling. Moreover, *GSTM2* inhibits hepatic steatosis by affecting the capability to detoxify and function as an antioxidant. Some previous studies supported our results. Han et al. found that *GSTM2* expression was much higher in ovarian teratoma mesenchymal stem cell-like cells than in normal cells and that p38 was highly inhibited in the teratomas^43^. Some early references also described the function of GSTs in regulating the activity of kinases such as ASK1 via protein-protein interactions^14,44–46^. Currently, the effect of ASK1-p38/JNK signalling on NAFLD and NASH has attracted the attention of researchers. It is important to note that there are currently some negative results of ASK1-related small molecule inhibitors (such as CFLAR-mimicking peptide) in clinical trials. Some studies have observed that these small molecule inhibitors may promote inflammation and amplify NASH liver injury^47^. Our findings may provide new insights into the role of ASK1 in NAFLD and contribute to the development of related molecular agents.

We also investigated the effect of *GSTM2* on LD growth. Inhibition of *GSTM2* resulted in a significant increase in the diameter of LDs formed by cells in a fatty acid-rich environment, and thus *GSTM2* appears to affect the LD growth process. According to previous studies, LD growth depends on lipid synthesis genes such as DGAT2 and GPAT4, and the key to the growth of LDs is the recruitment of DGAT2 and GPAT4 to the LD surface^18^. We inhibited the expression of *GSTM2* and found that recruitment of DGAT2 and GPAT4 to the surface of LDs was increased. Because hydrogen peroxide treatment did not significantly affect the LD diameter, intracellular ROS may have minimal effects on LD growth. We further examined whether ASK1 signalling affects the LD growth process. When cells were treated with 400 mM palmitic acid or 5 μM GS-4997, the recruitment of DGAT2 and GPAT4 on the surface of LDs showed significant differences. When ASK1 was activated, DGAT2 and GPAT4 were recruited to the LD surface, whereas when ASK1 activity was inhibited, recruitment of GPAT4 to the surface of LDs was reduced. Therefore, ASK1 may affect the growth of LDs by affecting the recruitment of DGAT2 and GPAT4 to the LD surface. No previous studies reported the effect of GSTM2 or ASK1-p38/JNK signalling on LD growth. This is the first study to demonstrate that ASK1-p38/JNK regulated LD growth by promoting DGAT2 and GPAT4 targeting to the surface of LDs. We did not investigate the mechanism of this regulatory role; therefore, further studies are needed.

**Fig. 7:**
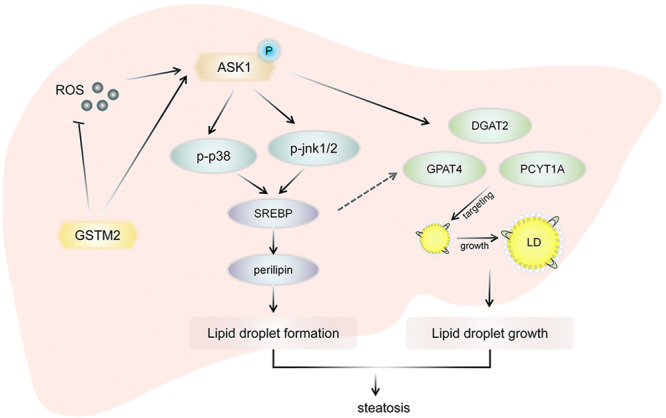
GSTM2 protects against the development of hepatic steatosis via its antioxidant and suppressing ASK1-p38/JNK signaling. GSTM2 was highly up-regulated in hepatic steatosis tissues. As the member of phase II drug metabolizing enzymes, GSTM2 could detoxify the toxicity of metabolic products such as ROS. ROS could enhance the inflammation and fibrosis progression. Furthermore, GSTM2 interacted with ASK1 directly and suppressed ASK1 phosphorylation. Therefore, GSTM2 down-regulated expression of SREBP and perilipins to inhibit the LD accumulation. ASK1 signaling also regulated the expression level of DGAT2, GPAT4 and PCYT1A and the recruitment of DGAT2 and GPAT4 on LD surface to modulate the LD growth. In conclusion, GSTM2 protects against the hepatic steatosis of NAFLD via its antioxidant and suppressing ASK1-p38/JNK signaling.

In summary, we identified *GSTM2* as a protective factor against hepatic steatosis. *GSTM2* interacted with ASK1 and suppressed its phosphorylation and the activation of downstream p38-JNK signalling. Moreover, GSTM2 plays an important role in detoxification and antioxidant activities, which protect tissues against injury induced by drugs or oxidative stress (Fig. 7). *GSTM2* showed a therapeutic effect on hepatic steatosis, providing a potential strategy for the clinical treatment of NAFLD.

## Materials and methods

### Animals and cell lines

*GSTM2* knockout mice were prepared by Cyagen Bioscience, Inc. (Santa Clara, CA, USA). The C57/BL-6 mice were used for the knockout construction. One single-base deletion was induced in exon3 of the GSTM2 (NM_008183.3) gene using the TALEN method. All mice were housed in a normal environment and provided with food and water. The 6–8 week ages male mice were used in this study. All experimental protocols were approved by the Ethics Committee of Huazhong Agricultural University. The HepG2 cell line was gifted by Xianghua Yan’s lab (Huazhong Agricultural University).

### Ethics approval and consent to participate

All mouse were housed in a normal environment provided with food and water. The methods were performed in accordance with the approved guidelines from Huazhong Agricultural University, and scientific, ethical and legal principles of the Hubei Regulations for the Administration of Affairs Concerning Experimental Animals. All experimental protocols were approved by the Ethics Committee of Huazhong Agricultural University.

### Antibodies

The following rabbit polyclonal antibodies were used: anti-GSTM2 (#ab125,102, Abcam, Cambridge, UK, 1:1000), anti-GSTM2 (#A13,496, ABclonal, Wuhan, China, 1:2000), anti-GAPDH (#CSB-PA00025A0Rb Flarebio Biotech LLC, College Park, MD, USA, 1:1000), anti-CIDEC (#D222114, Sangon Biotech, Shanghai, China, 1:1000), anti-CIDEC (#12287-1-AP, Proteintech, Rocky Hill, NJ, USA, 1:1000), anti-ADRP/perilipin2 (#15294-1-AP, Proteintech), anti-DGAT2 (#bs-12,998R, BIOSS, Beijing, China), anti-DGAT2 (#A13,891, ABclonal, Wuhan, China, 1:2000), anti-GPAT4 (#bs-15,587R, BIOSS), anti-SREBP1 (#14,088-1-AP, Proteintech), and anti-PCYT1A (#bs-11306R, BIOSS), anti-PCYT1A (#A5935, ABclonal, Wuhan, China, 1:1000). The mouse monoclonal antibody used was anti-tubulin (#GB13017-2, Servicebio, Wuhan, China, 1:1000). The following secondary antibodies were used: Alexa Fluor 555-labelled donkey anti-rabbit IgG (H+L) (#A0453, Beyotime, Shanghai, China, 1:200), horseradish peroxidase (HRP)-labelled goat anti-rabbit IgG (H+L) (#GB23303-1, Servicebio, 1:8000), HRP-labelled goat anti-mouse IgG (H+L) (#GB23301, Servicebio, 1:8000), and HRP-conjugated goat anti-rabbit IgG (#D110,058, Sangon Biotech, 1:8000).

### Plasmid DNA construction

Full-length coding sequences encoding *GSTM2* (NM_001142368.1) and PCYT1A (NM_005017.3) were amplified using a cDNA library of HepG2 cells and then subcloned into the pcDNA3.1 vector (gifted from Prof. Dequan Xu’s lab, Huazhong Agricultural University) and pEGFP-C1 and pmCherry-C1 vectors (gifted from Prof. Xianghua Yan’s lab, Huazhong Agricultural University). Sequences of the primers used for the *GSTM2* RNAi experiment were referred to study of Huenchuguala^48^. The sequences were subcloned into the pSuperior.puro vector for the RNAi experiment (gifted from Prof. Xianghua Yan’s lab).

### Cell culture and transfection

WT HepG2 or stably transfected cells (pSuperior.puro-GSTM2) were cultured in DMEM (HyClone, Logan, UT, USA) containing 10% foetal bovine serum (Clark Bioscience, Richmond, VA, USA), 100 U/mL penicillin, and 100 g/mL streptomycin in dishes at 37 °C and transfected with Lipo6000™ Transfection Reagent (#C0528, Beyotime). HepG2 cells were seeded onto a cell slide in a 24-well plate and transfected with plasmid vectors according to the transfection reagent instructions.

### HE staining and Oil Red O staining of histological sections

For HE staining, the liver tissues of three *GSTM2* knockout mice and three wild-type mice were divided and fixed in 4% paraformaldehyde for 12 h. Then the samples were performed the HE and Oil Red O staining by Servicebio (Wuhan, China) (http://www.servicebio.com/).

### ROS level detection

Cells were seeded into a 96-well black plate. ROS levels in the cells were detected using an ROS Assay Kit (#S0033, Beyotime) based on the DCFH-DA method. The ROS level was detected using a microplate spectrophotometer (PerkinElmer EnSpire, Waltham, MA, USA). The excitation and emission wavelengths were 488 and 525 nm, respectively. ROSup was used as an activator to increase the ROS level in cells, which was added to the wells at 1:1000 (v:v) with DMEM and incubated at 37 °C.

### Western blot and real-time PCR

Real-time PCR was performed using a Roche LightCycler 480 detection system (Roche, Basel, Switzerland). One negative control reaction in which the cDNA template was replaced with water was performed as a control. Each sample was amplified in triplicate, and the comparative Ct (ΔΔCt) value method was used for relative quantification. GAPDH was used as a reference gene. The Western blot method referred to our previous study^49^.

### Immunofluorescence assay

The immunofluorescence method referred to our previous study^49^. The confocal laser scanning microscope (Carle Zeiss, German) was used to observe the slide of cells. The images were analysed by ZEN pro software (Carle Zeiss, German), ImageJ and Photoshop CS6 (Adobe).

### Living cells workstation

The cells were seeded into a 35 mm confocal dish for 24 h culture and OA treatment, and then were washed 3 times with PBS and then added to the DMEM culture containing BODIPY (1:1000, v/v), followed by incubation for 20 min at 37 °C. The dish was placed in a living cell workstation, and intracellular LDs were observed using a 63 × oil immersion objective. The exposure time was set to 200 ms, the definitely focused 2.0 strategy was used, the interval of image capture was 15 s, and the duration of capture was 1 h. The data were analysed using ZEN pro and ZEN 2.3 (blue edition) software (ZEISS, Oberkochen, Germany).

### HFD and MCDD feeding experiment

Fifteen 4-week-old healthy male *GSTM2* knockout mice and 15 wild-type mice were selected for high-fat diet (HFD) feeding. The mice were divided into five groups of 3 individuals each. The mice were sacrificed on days 0, 5, 10, 20, and 30 after the start of HFD feeding and then liver tissues were collected. The formula of the HFD was ordinary mixed diet (88.5%), lard (10%), cholesterol (1%), and pig bile salt (0.5%). Six-week-old male mice were divided into two groups and fed an MCDD. The tissue collection was conducted as described above.

### Triglyceride content detection

The TG content of the liver tissues was detected by fully automatic chemistry analyser (#Chemray 240, Rayto Life and Analytical Sciences Co.,Ltd., Shenzhen, China). The total protein concentration was detected by Enzyme Label Detector (#Epoch, BioTek Instruments, Inc., headquartered in Winooski, VT, USA). The TG content data was normalized by protein concentrations, mmol/g.

### Plasmid and inhibitor injection

The *GSTM2* overexpression or control plasmid (200 μg) was mixed with HiGene I (14.4 μL) (#C1507, Applygen Technologies Inc., Beijing, China). Then the mixture was incubated at room temperature (RT) for 30 min. The mixture was injected intraperitoneally. By detection of *GSTM2* expression in liver, skeletal muscle, heart and brain, no significant change was observed except in liver tissues. In addition, 5 mg of GS-4997 (also called selonsertib; #S8292, Selleck, Shanghai, China), an ASK1 inhibitor, was mixed with 1 mL of dimethyl sulfoxide (DMSO) to prepare the 10 mM mother liquor. Then the mother liquor was diluted by normal saline for 80 μM working fluid, and the inhibitor was injected intraperitoneally.

### Statistics and reproducibility

All experiments were repeated three times. Data are shown as the mean ± SD. Student’s *t*-test was used for statistical comparisons. For experiments comparing more than two groups, one-way ANOVA and two-way ANOVA were used. *P* value < 0.05 was considered as statistically significant.

### Reporting summary

Further information on research design is available in the Nature Research Reporting Summary linked to this article.

## Supplementary information

Supplementary Information

Description of Additional Supplementary Files

supplementary movie S1

supplementary movie S2

Supplementary Data 1

Reporting Summary

## Data Availability

The source data for all the graphs in main figures in Supplementary Data 1. The data used to support the findings of this study are available from the corresponding author upon request.
