# Supplementary material for: Glutathione S-transferase Mu 2 inhibits hepatic steatosis via ASK1 suppression
Source: Commun Biol. 2022 Apr 6;5:326. doi: 10.1038/s42003-022-03251-w (PMC8986781; doi:10.1038/s42003-022-03251-w)
Supplement: Supplementary file 1 — Supplementary Information [file 42003_2022_3251_MOESM1_ESM.pdf]

Supplementary Information for

Glutathione S-transferase Mu 2 inhibits Hepatic Steatosis via ASK1 Suppression

Yi Jin[1,2], Yanjie Tan[1], Pengxiang Zhao[1], Yu Guo[1], Shilin Chen[1], Jian Wu[1,*], Zhuqing Ren[1,2*]

[1]Key Laboratory of Agriculture Animal Genetics, Breeding and Reproduction of the Ministry of Education & Key Laboratory of Swine Genetics and Breeding of the Ministry of Agriculture, College of Animal Science, Huazhong Agricultural University, Wuhan, Hubei, P. R. China, 430070

[2]Bio-Medical Center of Huazhong Agricultural University, Wuhan, Hubei, P. R. China, 430070

[*]Corresponding author. Address: College of Animal Science and Technology, Huazhong Agricultural University, Wuhan, 430070, P. R. China

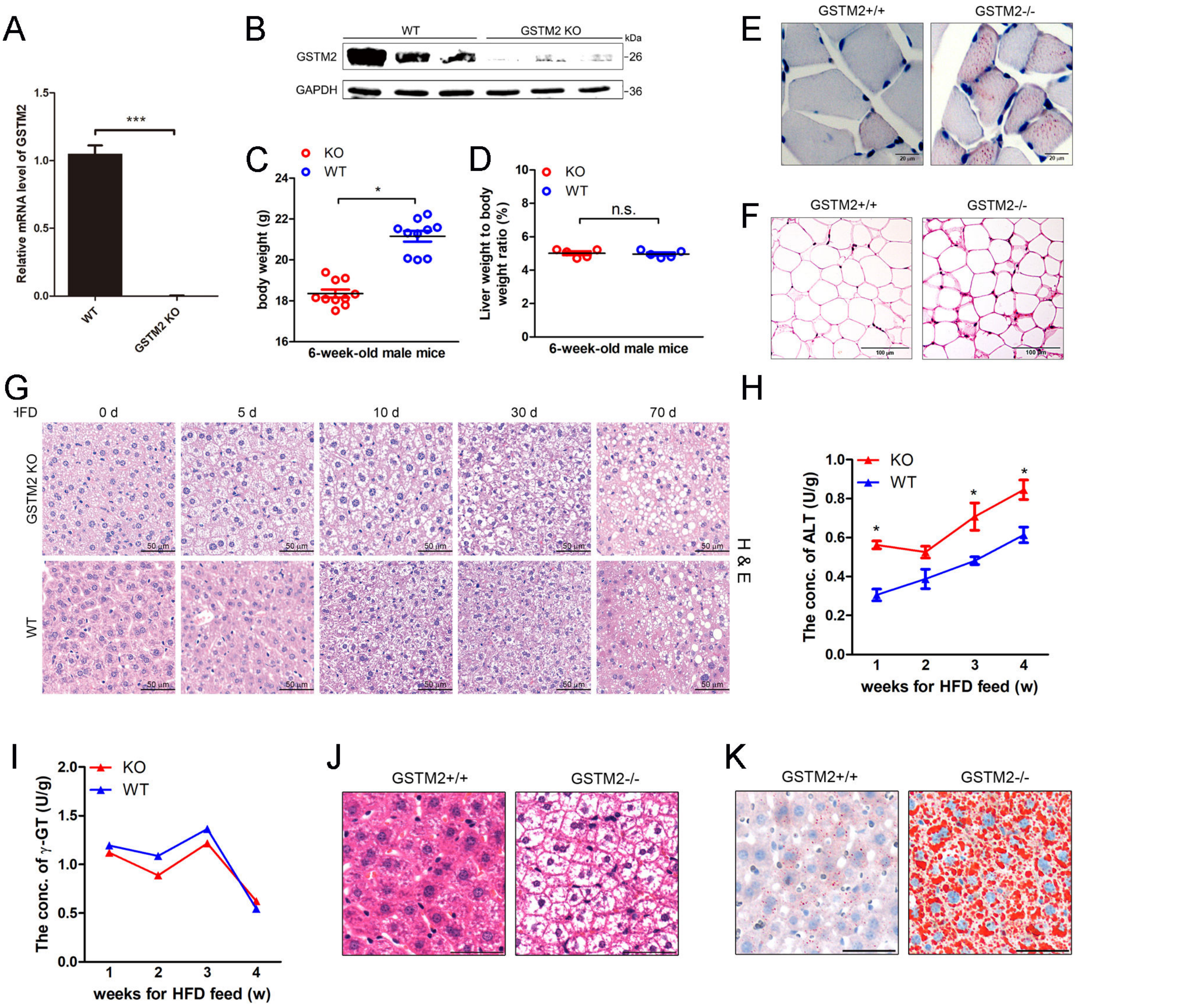

Supplementary Figure S1. (A, B) Detection of expression level of GSTM2 in GSTM2-knockout mice by qPCR and Western blot; ** $p < 0.01$. (C) Body weight detection of 6-week-old male GSTM2 KO and WT mice, *, $p<0.05$. (D) Detection of liver weight in body weight ratio of 6-week-old male GSTM2 KO and WT mice, n.s., no significant difference. (E) Oil red O staining analysis of skeletal muscle of GSTM2 KO and WT mice. (F) HE staining analysis of white adipose tissues of GSTM2 KO and WT mice. (G) HE staining analysis of liver samples of GSTM2 KO and control mice challenged with HFD at 0 d, 5 d, 10 d, 20 d, and 30 d. (H) HE staining analysis of white adipose tissues of GSTM2 KO and WT mice. (I) Detection of γ-GT concentration of GSTM2 KO and WT mice that were challenged to HFD feeding at 1, 2, 3 and 4 weeks. (J) HE staining analysis of liver tissues of GSTM2 KO and WT mice that were intraperitoneally injected with 200μM oleic acid. (K) Oil red O staining analysis of liver tissues of GSTM2 KO and WT mice that were intraperitoneally injected with 200μM oleic acid.

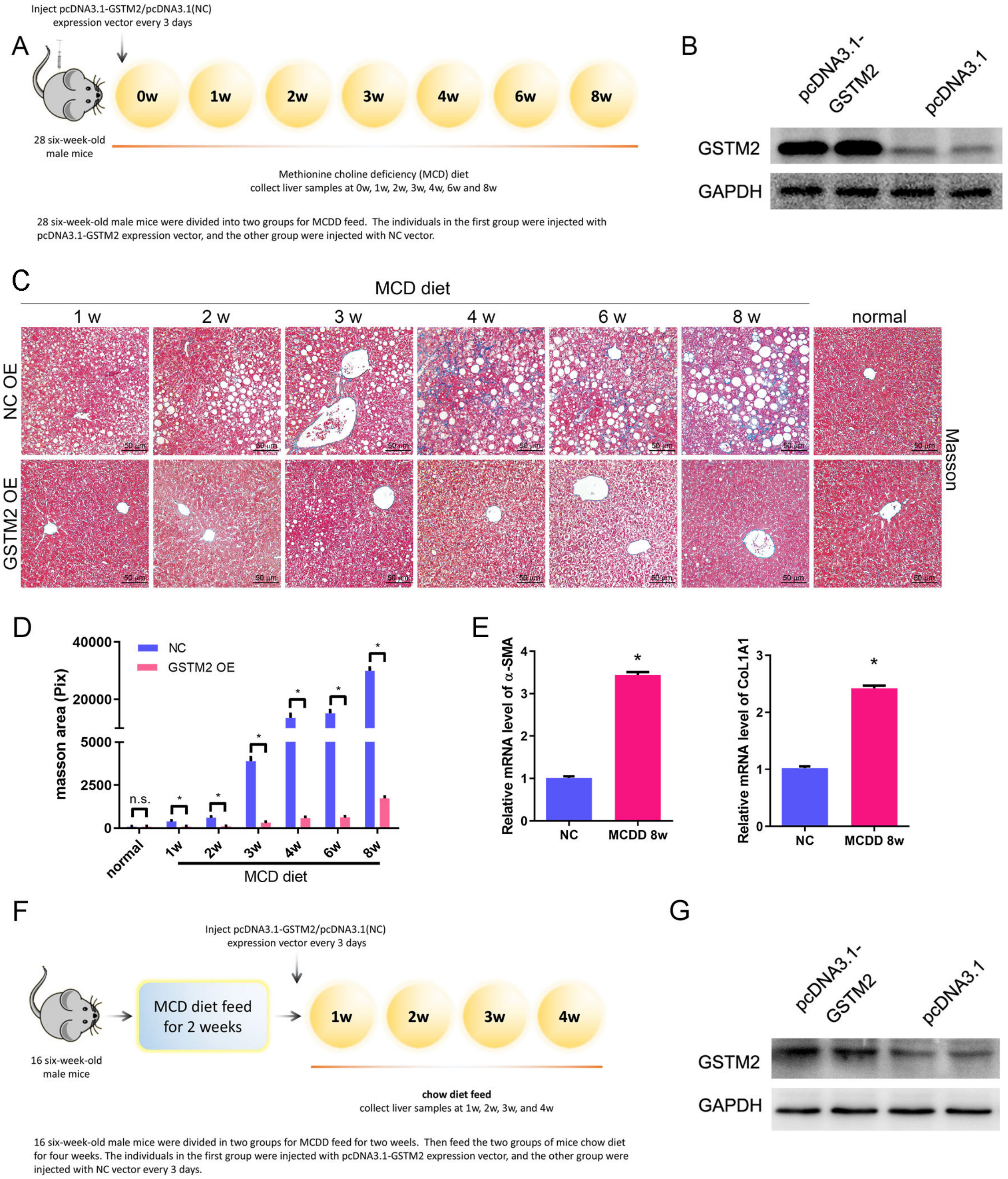

Supplementary Figure S2. A. Methionine choline deficiency (MCD) diet feeding was used to construct the NAFLD mouse model. Along with MCDD feeding, the mice were injected with GSTM2 overexpression vector (incubated with transfection reagent) or negative control vector every 3 days. The liver tissue samples were collected at 0w, 1w, 2w, 3w, 4w, 6w and 8w for the histological examinations. B. The detection of GSTM2 protein expression level of mouse model. (C) Masson staining analysis of liver samples of GSTM2 OE and control mice challenged with MCDD at 1 w, 2 w, 3 w, 4 w, 6 w, and 8 w. D. masson area analysis of C, *, $p<0.05$. E. qPCR detection of expression of fibrosis marker genes α-SMA and CoL1A1. F. The rescue experiment was performed

on the mouse model that were fed with MCDD for 2 weeks. The mice were injected with GSTM2 overexpression vector (incubated with transfection reagent) or negative control vector every 3 days. The liver tissue samples were collected at every 1 week for further histological examinations. G. The detection of GSTM2 protein expression level of mouse model of the rescue experiment.

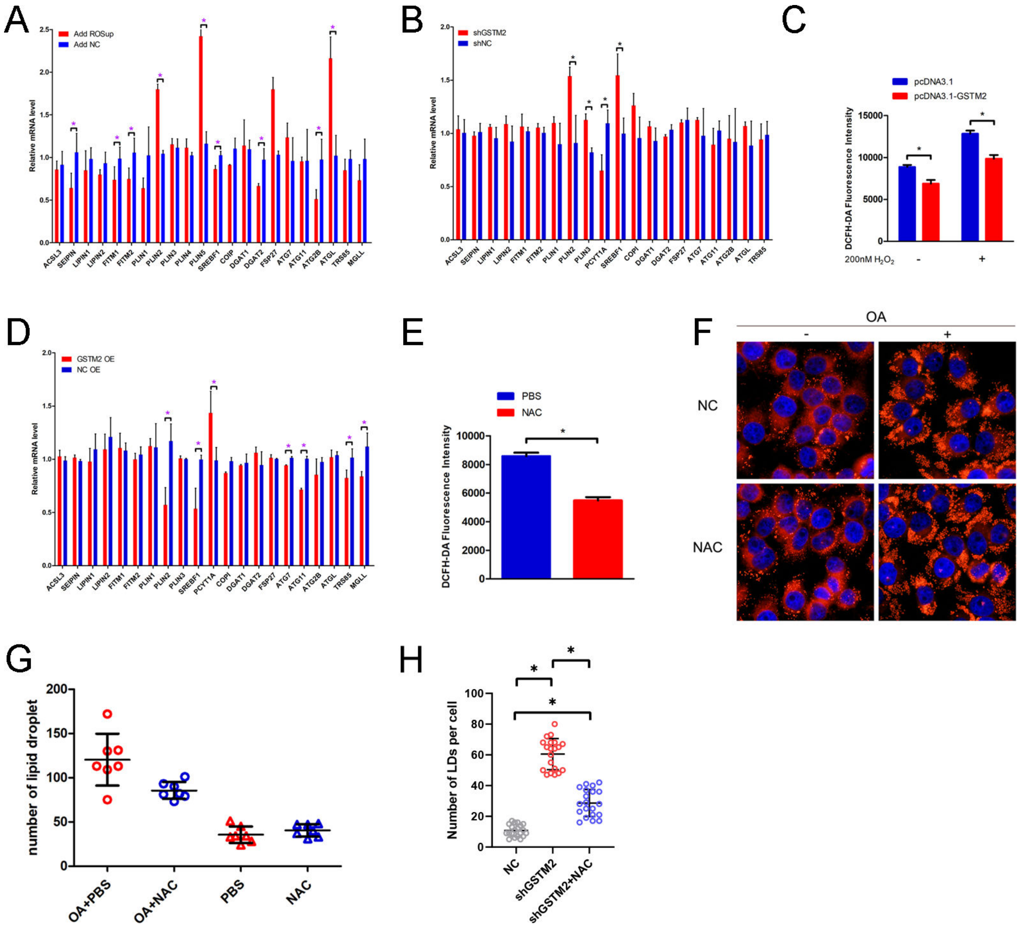

Supplementary Figure S3. A. The mRNA expression level of lipid droplet-related genes was detected by qPCR after the ROSup treatment. B. The mRNA expression level of lipid droplet-related genes was detected by qPCR after knocking down the expression of GSTM2. C. The cellular ROS level was detected in GSTM2-overexpressed cells and control cells in the presence of 200nM hydrogen peroxide or not, *, $p<0.05$ via DCFH-DA method. D. The mRNA expression level of lipid droplet-related genes was detected by qPCR after overexpressing GSTM2. E. The cellular ROS level was detected in cells treated with Nacetylcysteine (NAC). F. The cellular lipid droplets were stained by Nile Red in cells treated with NAC and control cells in the presence of oleic acid medium or not. G. The statistic of the number of lipid droplets in F. H. detection of LDs number in shGSTM2 cells and shGSTM2 cells with NAC treatment, *, $p<0.05$.

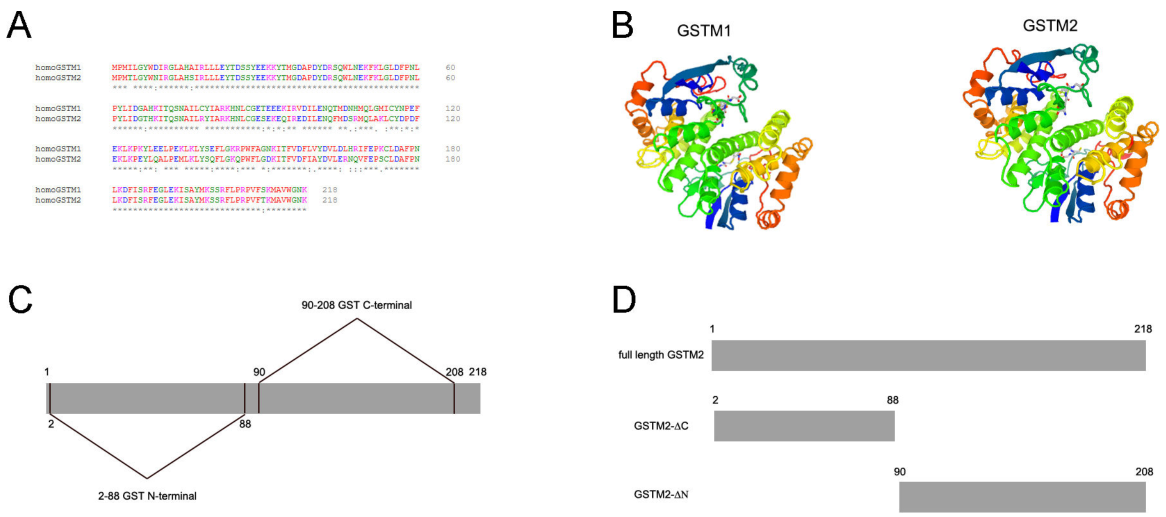

Supplementary Figure S4. A. The comparison of amino acid sequences between GSTM2 and GSTM1. B. The comparison of 3D structure between GSTM2 and GSTM1. C. The description of domains of GSTM2 protein. D. The GSTM2-ΔC and GSTM2-ΔN expression vector were constructed according to the GSTM2 domains.

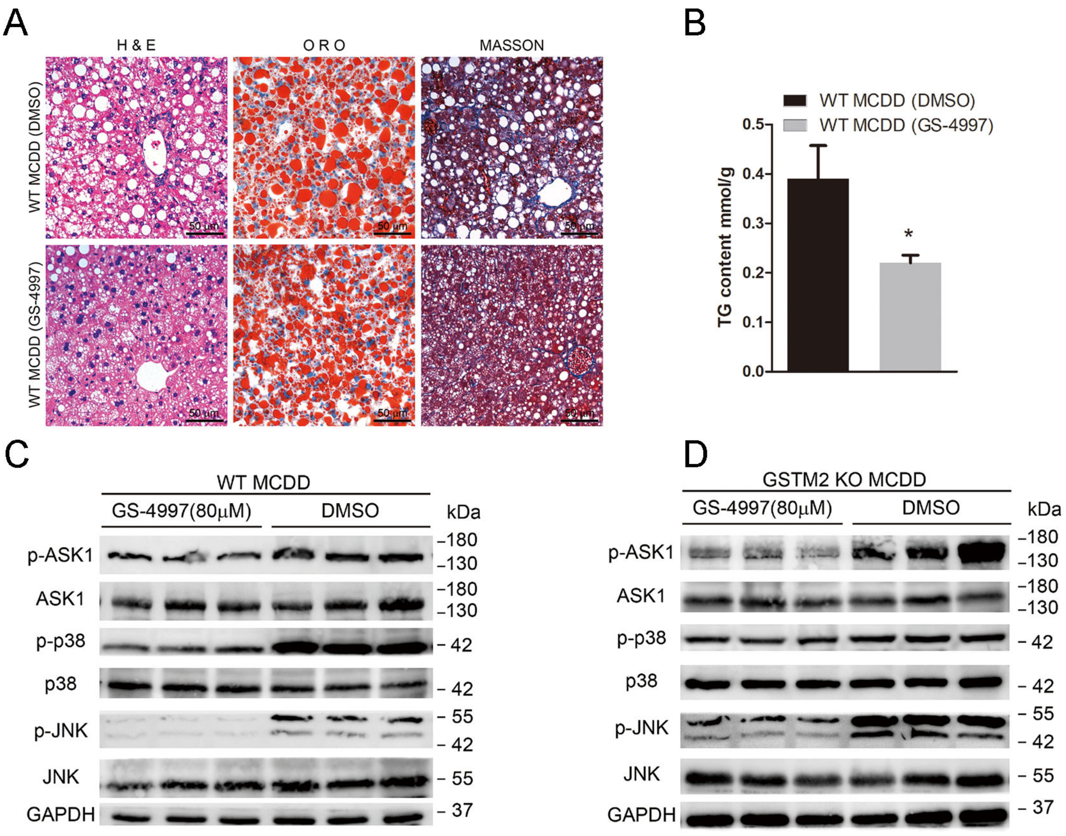

Supplementary Figure S5. A. WT mice were fed MCDD and treated with GS-4997 or DMSO by intraperitoneal injection. Then hepatic fat and fibrosis level were detected by HE, ORO and Masson

staining. B. TG content examination of WT mice fed MCDD with GS-4997 or DMSO treatment. C. Investigation of protein levels of ASK1 signalling pathway in WT mice fed MCDD with GS-4997 or DMSO treatment by Western blot. D. Grey value analysis of C by ImageJ software. E. Investigation of protein levels of ASK1 signalling pathway in GSTM2 KO mice fed MCDD with GS-4997 or DMSO treatment by Western blot. F. Grey value analysis of E by ImageJ software.

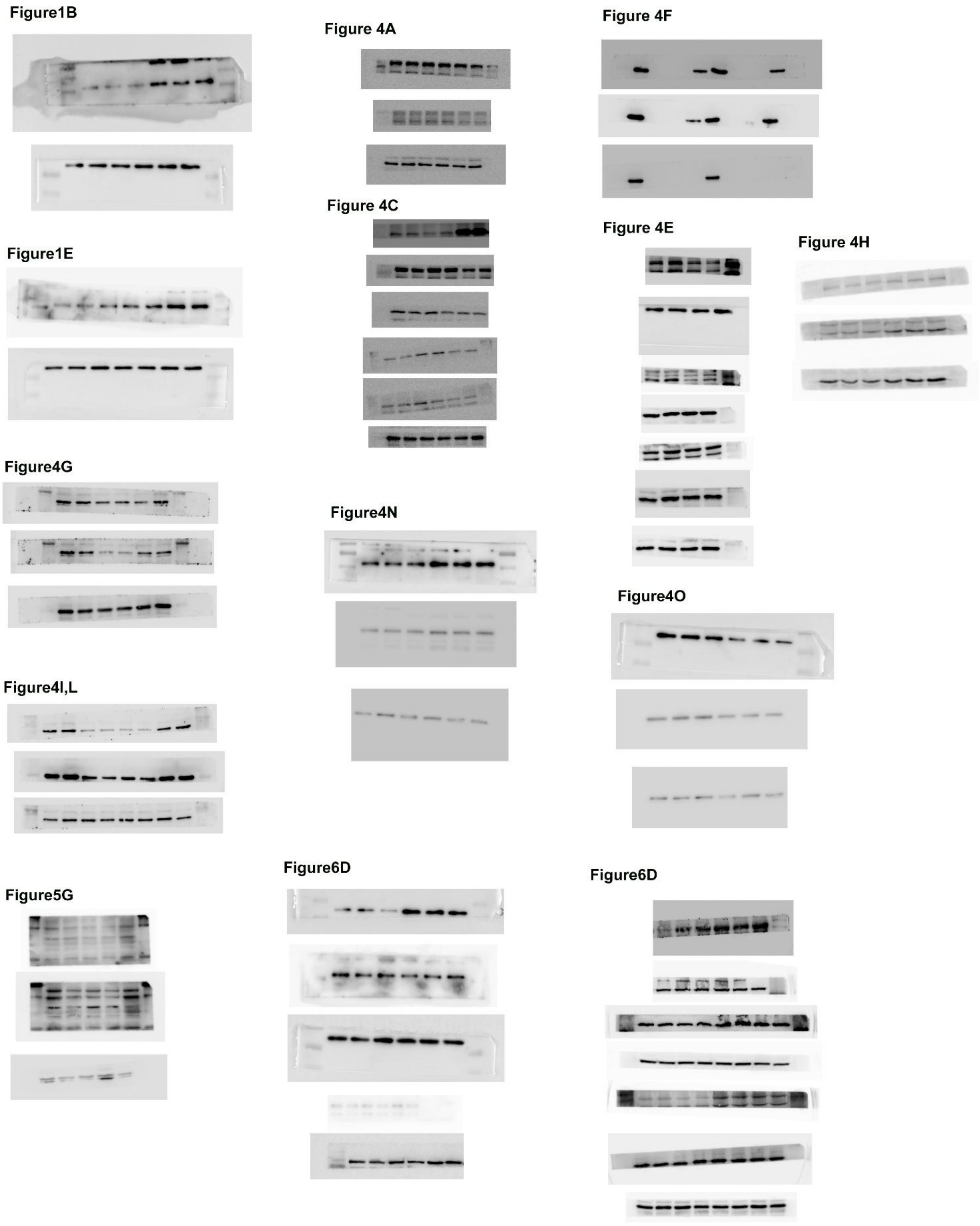

Supplementary Figure S6. uncropped western blots.
