# Supplementary material for: Glutathione S-transferase Mu 2 inhibits hepatic steatosis via ASK1 suppression
Source: Commun Biol. 2022 Apr 6;5:326. doi: 10.1038/s42003-022-03251-w (PMC8986781; doi:10.1038/s42003-022-03251-w)
Supplement: Supplementary file 2 — Description of Additional Supplementary Files [file 42003_2022_3251_MOESM2_ESM.pdf]

Description of Additional Supplementary Files

File name: Supplementary Movie S1
Description: Observation of the movement of lipid droplets in GSTM2 knockdown cells by living cell station. The GSTM2 knockdown cells were seeded in 35 mm confocal dish for 24-hour culture, and the oleic acid culture medium was used for 12 hours. The cells were washed for 3 times with PBS and then added to the DMEM culture containing BODIPY (1:1000, v/v) to incubate for 20 minutes at 37℃. The dish was placed in a living cell workstation, and the intracellular lipid droplets were observed by 63×oil immersion objective. The exposure time was set to 200 milliseconds, the definitely focus 2.0 strategy is used, the capturing interval was 15 seconds, and the duration of capturing was 1 hours. The LD-LD contact events were pointed using arrows.

File name: Supplementary Movie S2
Description: Observation of the movement of lipid droplets in NC cells by living cell station.

File name: Supplementary Data 1
Description: Source data for all the graphs in main figures.
