# Supplementary material for: Glutathione S-transferase Mu 2 inhibits hepatic steatosis via ASK1 suppression
Source: Commun Biol. 2022 Apr 6;5:326. doi: 10.1038/s42003-022-03251-w (PMC8986781; doi:10.1038/s42003-022-03251-w)
Supplement: Supplementary file 6 — Reporting Summary [file 42003_2022_3251_MOESM6_ESM.pdf]

nature portfolio

Corresponding author(s): Zhuqing Ren

Last updated by author(s): 2022-02-11

Reporting Summary

Nature Portfolio wishes to improve the reproducibility of the work that we publish. This form provides structure for consistency and transparency in reporting. For further information on Nature Portfolio policies, see our Editorial Policies and the Editorial Policy Checklist.

Statistics

For all statistical analyses, confirm that the following items are present in the figure legend, table legend, main text, or Methods section.

n/a	Confirmed	
☐	☒	The exact sample size (*n*) for each experimental group/condition, given as a discrete number and unit of measurement
☐	☒	A statement on whether measurements were taken from distinct samples or whether the same sample was measured repeatedly
☐	☒	The statistical test(s) used AND whether they are one- or two-sided<br>*Only common tests should be described solely by name; describe more complex techniques in the Methods section.*
☒	☐	A description of all covariates tested
☐	☒	A description of any assumptions or corrections, such as tests of normality and adjustment for multiple comparisons
☐	☒	A full description of the statistical parameters including central tendency (e.g. means) or other basic estimates (e.g. regression coefficient) AND variation (e.g. standard deviation) or associated estimates of uncertainty (e.g. confidence intervals)
☐	☒	For null hypothesis testing, the test statistic (e.g. *F*, *t*, *r*) with confidence intervals, effect sizes, degrees of freedom and *P* value noted<br>*Give P values as exact values whenever suitable.*
☒	☐	For Bayesian analysis, information on the choice of priors and Markov chain Monte Carlo settings
☒	☐	For hierarchical and complex designs, identification of the appropriate level for tests and full reporting of outcomes
☒	☐	Estimates of effect sizes (e.g. Cohen's *d*, Pearson's *r*), indicating how they were calculated

Our web collection on statistics for biologists contains articles on many of the points above.

Software and code

Policy information about availability of computer code

Data collection	This study did not involve software and code for data collection.
Data analysis	We used standard software throughout the manuscript (GraphPad Prism version 6.07) that is publicly available.

For manuscripts utilizing custom algorithms or software that are central to the research but not yet described in published literature, software must be made available to editors and reviewers. We strongly encourage code deposition in a community repository (e.g. GitHub). See the Nature Portfolio guidelines for submitting code & software for further information.

Data

Policy information about availability of data

All manuscripts must include a data availability statement. This statement should provide the following information, where applicable:

- Accession codes, unique identifiers, or web links for publicly available datasets
- A description of any restrictions on data availability
- For clinical datasets or third party data, please ensure that the statement adheres to our policy

All data are available within the Article or Supplementary Information. The data used to support the findings of this study are available from the corresponding author upon request.

Field-specific reporting

Please select the one below that is the best fit for your research. If you are not sure, read the appropriate sections before making your selection.

- [x] Life sciences
- [ ] Behavioural & social sciences
- [ ] Ecological, evolutionary & environmental sciences

For a reference copy of the document with all sections, see nature.com/documents/nr-reporting-summary-flat.pdf

Life sciences study design

All studies must disclose on these points even when the disclosure is negative.

Sample size	Sample sizes were based on the experience of the authors with molecular and invivo studies as published in many studies.<br>For animal models, experiments were designed to detect differences between treatment groups or genotype-dependent effects at 80% power.<br>Sample sizes may vary depending on animal availability.<br>Sample size for cell based assays were determined based on sample availability
Data exclusions	No data was excluded from the analyses.
Replication	All experiments were repeated with reproducibility. The replication number for each experiment is indicated in the legend of the corresponding figure.
Randomization	For in vitro studies, the cells from each cell line required for all tested conditions were pooled, equal number of cell were then seeded and stimulated/treated randomly. For in vivo studies, the animal were allocated to experimental groups to ensure equal litter/sex/age across groups
Blinding	For in vitro experiments, investigators were not blinded to group allocation during data collection and analysis. For in vivo experiments, treatments/genotypes were not disclosed to investigators generating quantitative readouts during data collection but investigators were not blinded during data analysis.

Reporting for specific materials, systems and methods

We require information from authors about some types of materials, experimental systems and methods used in many studies. Here, indicate whether each material, system or method listed is relevant to your study. If you are not sure if a list item applies to your research, read the appropriate section before selecting a response.

Materials & experimental systems

n/a	Involved in the study	
☐	☒	Antibodies
☐	☒	Eukaryotic cell lines
☒	☐	Palaeontology and archaeology
☐	☒	Animals and other organisms
☒	☐	Human research participants
☒	☐	Clinical data
☒	☐	Dual use research of concern

Methods

n/a	Involved in the study	
☒	☐	ChIP-seq
☒	☐	Flow cytometry
☒	☐	MRI-based neuroimaging

Antibodies

Antibodies used	The following rabbit polyclonal antibodies were used: anti-GSTM2 (#ab125102, Abcam, Cambridge, UK), anti-GAPDH (#CSB-PA00025A0Rb Flarebio Biotech LLC, College Park, MD, USA), anti-CIDEC (#D222114, Sangon Biotech, Shanghai, China), anti-CIDEC (#12287-1-AP, Proteintech, Rocky Hill, NJ, USA), anti-ADRP/perilipin2 (#15294-1-AP, Proteintech), anti-DGAT2 (#bs-12998R, BIOSS, Beijing, China), anti-GPAT4 (#bs-15587R, BIOSS), anti-SREBP1 (#14088-1-AP, Proteintech), and anti-PCYT1A (#bs-11306R, BIOSS). The mouse monoclonal antibody used was anti-tubulin (#GB13017-2, Servicebio, Wuhan, China). The following secondary antibodies were used: Alexa Fluor 555-labelled donkey anti-rabbit IgG (H+L) (#A0453, Beyotime, Shanghai, China), horseradish peroxidase (HRP)-labelled goat anti-rabbit IgG (H+L) (#GB23303-1, Servicebio), HRP-labelled goat anti-mouse IgG (H+L) (#GB23301, Servicebio), and
Validation	All antibodies used are commercially available and validated by the manufacturers, as indicated on the respective websites of each commercial vendor.

Eukaryotic cell lines

Policy information about cell lines

Cell line source(s)	HepG2 (ATCC)

Authentication	The HepG2 cell line was gifted by Xianghua Yan's lab (Huazhong Agricultural University).
Mycoplasma contamination	All cell lines were tested to be free of mycoplasma contamination.
Commonly misidentified lines (See ICLAC register)	No commonly misidentified cell lines were used in the study.

Animals and other organisms

Policy information about studies involving animals; ARRIVE guidelines recommended for reporting animal research

Laboratory animals	GSTM2 knockout mice were prepared by Cyagen Bioscience, Inc. (Santa Clara, CA, USA). One single-base deletion was induced in exon3 of the GSTM2 (NM_008183.3) gene using the TALEN method. All mice were housed in a normal environment and provided with food and water. All experimental protocols were approved by the Ethics Committee of Huazhong Agricultural University. Fifteen 4-week-old healthy male GSTM2 knockout mice and 15 wild-type mice were selected for high-fat diet (HFD) feeding. The mice were divided into five groups of 3 individuals each. The mice were sacrificed on days 0, 5, 10, 20, and 30 after the start of HFD feeding and then liver tissues were collected. The formula of the HFD was ordinary mixed diet (88.5%), lard (10%), cholesterol (1%), and pig bile salt (0.5%). Six-week-old male mice were divided into two groups and fed an MCDD. The tissue collection was conducted as
Wild animals	The study did not involve wild animals.
Field-collected samples	The study did not involve field collected animals.
Ethics oversight	Animal experiments were performed under the guidelines of the Ethics Committee of Huazhong Agricultural University.

Note that full information on the approval of the study protocol must also be provided in the manuscript.
